# Reduction of Capacity Fading in High-Voltage NMC Batteries with the Addition of Reduced Graphene Oxide

**DOI:** 10.3390/ma15062146

**Published:** 2022-03-15

**Authors:** Yahya M. Alqahtani, Quinton L. Williams

**Affiliations:** Department of Physics and Astronomy, Howard University, 2355 6th St., NW, Washington, DC 20059, USA; yahya.alqahtani@bison.howard.edu

**Keywords:** electric vehicle (EV), graphene, reduced graphene oxide, NMC811, cathode, Li-ion batteries

## Abstract

Lithium-ion batteries for electric vehicles (EV) require high energy capacity, reduced weight, extended lifetime and low cost. EV manufacturers are focused on Ni-rich layered oxides because of their promising attributes, which include the ability to operate at a relatively high voltage. However, these cathodes, usually made with nickel–manganese–cobalt (NMC811), typically experience accelerated capacity fading when operating at a high voltage. In this research, reduced graphene oxide (rGO) is added to a NMC811 cathode material to improve the performance in cyclability studies. Batteries made with rGO/NMC811 cathodes showed a 17% improvement in capacity retention after 100 cycles of testing over a high-voltage operating window of 2.5–4.5 V.

## 1. Introduction

Nickel–manganese–cobalt (NMC811) cathodes, also known as high-Ni or Ni-rich cathodes, are increasingly becoming popular due in part to the enormous technological advances made in the design and manufacture of electric vehicles (EV) [1]. This has been driven by the development of low-cost NMC811 batteries with high energy density. The high energy density of Ni-rich cathodes is of paramount importance for producing high energy density Li-ion batteries to increase the range of EVs. Intense interest in investigations by scientists and engineers has emerged as they attempt to optimize rate capability and cost vs. performance efficiency in Ni-rich NMC811 batteries. The time that it takes to recharge an EV is a critical component for advancing EV technologies so as to imitate the relatively short (∼5 min) refueling time of fossil fuel transportation vehicles. However, reducing the charging time may also lead to the diminution of safety parameters. In other words, utilizing Ni-rich technologies to further enhance the levels of EV performance must also be aligned with higher levels of safety for the consumer, with consumer safety being non-negotiable [2,3].

Ni-rich layered-transition-metal mixed oxides are being investigated intensely because of their ability to deliver higher power ratings with higher energy densities and reduced Co content. The practical upper voltage limit for NMC811 is ca. 4.3 V vs. Li/Li${}^{+}$; for LiFePO${}_{4}$ (LFP), it is ca. 3.5 V vs. Li/Li${}^{+}$ [4]. The improvements over LFP batteries derive from NMC811’s higher lithium diffusion rate and electron mobility. The layered crystal structure framework of NMC811 allows Li${}^{+}$ ions to have 2-dimensional movement as opposed to the restricted 1-dimensional movement in the ordered olivine crystal structure framework of LFP [5,6,7]. It has been reported that the lithium diffusion coefficient for NMC811 is 10,000 times faster than that of LFP, and the electron mobility is 1000 times faster [8]. The earliest Li-ion batteries were designed with a cathode made of lithium cobalt oxide (LiCoO${}_{2}$), while the anode was constructed from graphitic carbon material. As alternatives, cobalt was replaced by other metals, such as aluminum, manganese and nickel, leading to both the NMC811 [Li(Ni,Mn,Co)O${}_{2}$] and NCA [Li(Ni,Co,Al)O${}_{2}$] cathodes that are utilized in EVs and other transportation applications. Both aluminum and manganese are essentially inactive materials that function as stabilizing agents to optimize safety characteristics and properties while helping to increase the number of charge- and discharge cycles of NMC and NCA batteries.

Increasing the nickel content in the cathode material leads to an increase in the volume of lithium that can be intercalated into the cathode, which results in an increase in energy density. From a business perspective, replacing cobalt cathodes with nickel-rich cathodes is an important alternative because cobalt manufacturing has been blighted by human rights issues in production due to suspect mining practices. Additionally, the cobalt industry is very volatile and there are questions surrounding the sustainability of cobalt. Ni-rich active material is widely viewed as being more beneficial to the Li-ion battery industry, as nickel deposits are widely available worldwide and can be mined and produced in significantly larger quantities in comparison to cobalt. Moreover, the price of nickel is protected because this material is part of the large alloy and steel global markets. Many cutting-edge, commercially available cathodes being used in NMC811 batteries now contain up to around 80% nickel (metal-site basis).

While there are many positive properties of Ni-rich cathodes, there are also challenges in terms of thermal and cycling stability [9,10]. Transition metal ion dissolution occurs when the Ni-rich cathode is charged to a high-voltage, which results in a loss of capacity in the NMC811 battery [11]. The Li/Ni disorder is another known issue, but it can be reduced as the nickel content is enriched. Additionally, the oxygen evolution results from the thermodynamic instability of the bulk Ni-rich layered oxide material and may be slowed kinetically by long oxygen diffusion paths, surface coating, core-shell structures and Ni-concentration gradient materials [12]. For characterization purposes, cycle testing is an excellent means to observe the performance degradation. Finally, while problems exist during the overcharging process, it can also be beneficial for the NMC811 battery, as it tends to reduce voltage decay because battery overcharging dissolves nickel oxides and other passivating surface layers [13].

Ni-rich layered high-performance cathodes capable of operating at high voltage are viewed as next generation materials, due to their cost-effectiveness and high discharge capacity ∼180–230 mAh g${}^{-1}$. However, Ni-rich cathode materials are fettered by serious challenges that are countering their large-scale integration with the evolving electric transportation marketplace, where high standards are being required of future generation batteries for EVs. High-voltage applications and other large electrical appliances designed for the industry are demanding energy density properties of >400 Wh kg${}^{-1}$ or >500 Wh L${}^{-1}$. The cathode performance demanded by the EV version of the automotive industry is directed at Ni-rich NMC811 and NCA technologies in terms of functionality at high voltages.

The electrochemical performance of NMC811 during high-voltage operation is subjected to liquid electrolyte penetration limits and other limitations. The NMC811 nanoparticles’ morphology results in a heterogeneous state-of-charge (SOC), which is distributed throughout particles that are found in a cathode’s active material and results in particles being subjected to inhomogeneous aging. Additionally, charging NMC811 to high SOCs has an adverse structural impact due to the severe anisotropic volumetric changes in the NMC811 unit cell as the *c* lattice parameter collapses for x(Li) < 0.5 [14]. Furthermore, lithium extraction causes compositional heterogeneity (or spatial gradients) within the active material particle, which is a consequence of the non-uniform distributions of the interfacial chemical products, surface phases, and reaction sites. Spatial gradients in the lithium concentration induce internal strains, which are manifested as particle cracking and intergranular fracture [15]. Unreacted lithium ingredients, such as the oxide form of Li${}_{2}$O, are sometimes located on the surfaces of the active materials during both low- and high-voltage operation. This is primarily due to excessive quantities of lithium, which is needed to create crystalline Ni-rich layered materials [16].

The use of nickel-rich NMC811 as a cathode material for high-energy Li-ion batteries is highly desirable; however, operating at high-voltage levels (>4.3 V) is known to significantly accelerate capacity fading caused by side reactions and structural instability. Investigations of the performance loss in batteries have identified three main categories in the aging process: the loss in the inventory of lithium that is available to do work, loss of active material, and an increase in the internal resistance of a battery [17,18]. Surface coating NMC811 with Al${}_{2}$O${}_{3}$ using atomic layer deposition (ALD) has been used to reduce charge transfer impedance growth [19]. Research has shown that NMC811 coated with Al${}_{2}$O${}_{3}$ improves the capacity retention and yields ∼40% capacity fading at 2C for operation in a voltage window of 3.0–4.8 V vs. Li${}^{+}$/Li [20].

Herein, we examine the effect of using reduced graphene oxide as an additive to NMC811. Graphene, a 2D material, has outstanding electrical, mechanical and thermal properties and a high surface area (theoretical value of 2630 $m^{2}\;g^{-1}$) [21]. The reduction in graphene oxide has been widely reported in the literature, and it is a commercially available product [22,23,24]. Our work demonstrates that by incorporating reduced graphene oxide as an additive to NMC811, it is possible to significantly reduce capacity fading at 2C operation in a voltage window of 2.5–4.5 V vs. Li${}^{+}$/Li. This facile, low-cost process for preparing NMC811 cathode material with the addition of commercially available reduced graphene oxide results in a capacity degradation that is approximately half of the capacity reduction that is reported for batteries fabricated with the use of employing the expensive ALD process for surface coating Al${}_{2}$O${}_{3}$ onto NMC811 for high-voltage operation.

## 2. Materials and Methods

Chemically reduced graphene oxide (rGO) powder (D10 1–3 μm particle size, Graphene) was combined with carbon black (CB) powder (Super C45 carbon black, MTI Corporation, Richmond, CA, USA) to form a dry powder mixture of carbon black/reduced graphene oxide (CB/rGO). The CB/rGO powder was then mixed with Ni-rich NMC811 (${\mathrm{LiNi}}_{0.8}{\mathrm{Mn}}_{0.1}{\mathrm{Co}}_{0.1}O_{2}$) powder (<0.5 μm particle size, >95%, Sigma-Aldrich, Inc., St. Louis, MO, USA) to form the active cathode material. Then, a N-methyl-2-pyrrolidone (NMP) solvent (MTI Corp.) and polyvinylidene fluoride (PVDF) binder (MTI Corp.) were introduced to form a slurry. The mixing ratio for NMC811:PVDF:CB:rGO was 80:10:5:5. For the pristine NMC811 control/reference batteries, the NMC811:PVDF:CB mixing ratio was 8:1:1. The slurry was homogenized with a Vortex Genie 2 mixer (model G560, Scientific Industries, Inc., Bohemia, NY, USA) to produce a uniform 20 μm thick coating onto Al metal foil, using the doctor blade technique. The Al foil current collector was placed in a vacuum oven at 80 ${}^{\circ }C$ and allowed to dry for 24 h. Cathodes were pressed at a pressure of 10 MPa (model YLJ-24T, MTI Corp.) and then cut into 1.5 cm diameter discs using a precision disc cutter (model MSK-T-06, MTI Corp.) to form the working electrode for CR2032 coin-type half cells. The active material loading of the working electrode was ∼4.5–6.5 mg. A glovebox (MBraun LABmaster sp, $25{\;}^{\circ }C\pm 1{\;}^{\circ }C,O_{2}<0.1\;\mathrm{ppm},H_{2}O<0.1\;\mathrm{ppm}$) filled with high-purity argon (Ar, Airgas 99.999% purity) was used during the assembly of the coin cells. Pure metallic lithium metal chips (EQ-Lib-LiC60-300, 16 mm diameter and 0.6 mm thickness, 99.9% pure Li, MTI Corp.) were used as the anode for the half-cell batteries. A microporous separator film (Celgard 2400, 25 μm thickness) and 1M LiPF${}_{6}$ in 3:7 ethylene carbonate (EC)/ethyl methyl carbonate (EMC) (3:7 v/v) electrolyte (EQ-LBC3015B-LD, MTI Corp.) were used in the battery assembly. All chemicals and solvents were used as received.

The morphological features of the cathode material were studied using a scanning electron microscope (SEM; Phenom Pure 800-07882, FEI Company, Hillsboro, OR, USA). A battery test set (BT-2000, Arbin Instruments, College Station, TX, USA) was used to evaluate the electrochemical behavior of the assembled batteries in terms of the galvanostatic charge/discharge cycling and cyclic voltammetry (CV) over an operating voltage range of 2.5–4.5 V. The CV scan rate used was 0.1 mV s${}^{-1}$. All measurements were taken at ambient temperature.

## 3. Results and Discussion

Prior work by our group showed that graphene nanoplatelets (GNP), introduced as conductive additives in lithium–iron–phosphate composite cathode material, created a flexible three-dimensional conductive network through a plane-to-point connection with LiFePO${}_{4}$ nanoparticles [25]. Electrochemical tests showed that the LiFePO${}_{4}$/GNP cathode increased the specific discharge capacity and improved the battery performance at high C-rates due to enhanced electrochemical reactivity. This was attributed to more efficient electronic transport, which is facilitated by the ability of GNPs to bridge multiple LFP particles, owing to its larger surface area.

To begin this work, a comparison study of rGO/NMC811 batteries was completed for batteries prepared using 2wt%, 5wt% and 10wt% rGO to optimize the amount of rGO that would be used for this research. This comparison led to the decision of using 5wt% rGO to be used because it yielded the highest discharge capacity (216.48 mAh g${}^{-1}$) at 0.1C coupled with the lowest capacity fade (15%) after 100 cycles—see Table 1.

**Figure 1:**
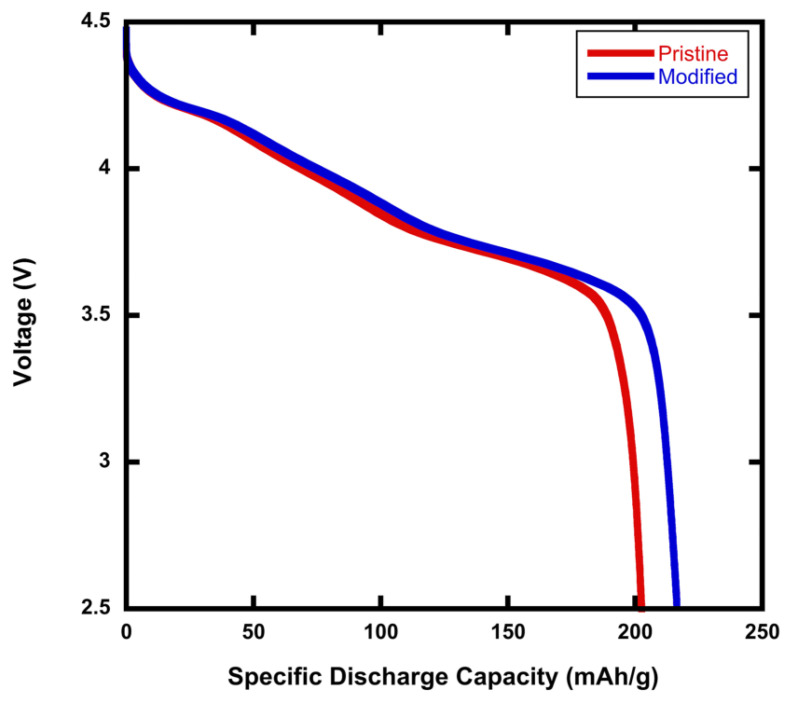
Initial discharge profiles for batteries made using pristine NMC811 and rGO/NMC811. In the plot legend, modified denotes rGO/NMC811 with 5wt% rGO. The rGO additive increases the discharge capacity by ∼5%. The measurement was conducted at 0.1C.

Figure 1 is a comparison of the specific discharge capacity for pristine NMC811 and modified rGO/NMC811 batteries—that is, NMC811 with 5wt% rGO added. The C-rate during testing was 0.1C. The specific discharge capacity of the battery fabricated with the rGO/NMC811 cathode was 216.48 mAh g${}^{-1}$, which is 5% higher than that of the battery made using pristine NMC811. For each rGO/NMC811 sample, a reference battery was made with pristine NMC811. In repeatability testing, the rGO/NMC811 batteries had a modest increase in the specific discharge capacity in comparison with their pristine NMC811 cathode references.

**Figure 2:**
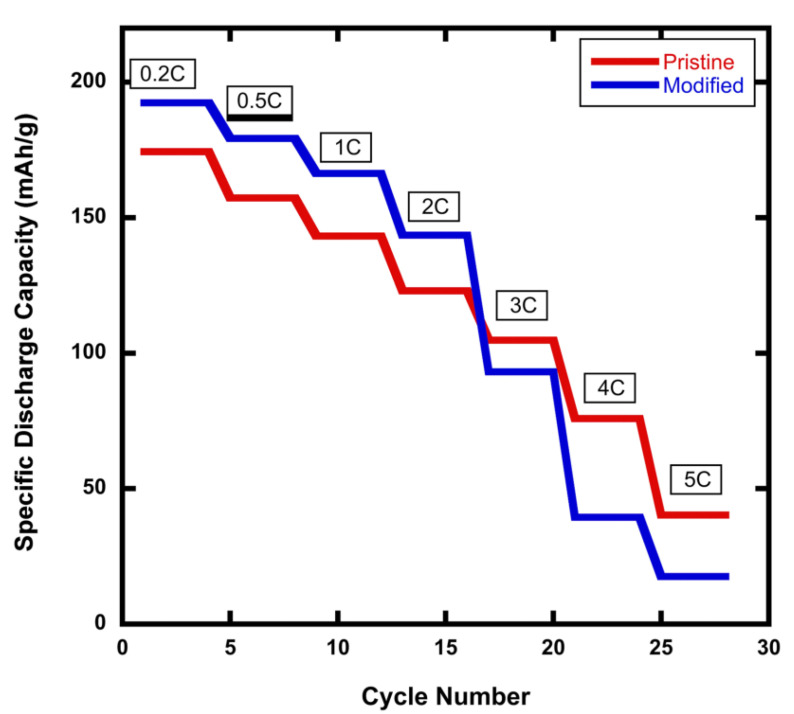
C-rate performance for batteries made with pristine NMC811 and modified rGO/NMC811 cathodes. The modified rGO/NMC811 battery has a higher specific discharge at C-rates < 3C. For C-rates > 3C, the pristine NMC811 battery experiences a higher specific discharge capacity.

Figure 2 shows the C-Rate performance profiles in the range between 0.2C and 5C for a comparison of the pristine NMC811 cathode versus the modified rGO/NMC811 cathode with 5wt% rGO. The modified rGO/NMC811 battery exhibits higher specific discharge capacity at C-rates below 3C. However, at high C-rates, the specific discharge capacity of the rGO/NMC811 battery cathode exhibits a significant decline in its C-rate performance. It was found that 3C is the diffusion-limited C-rate for the modified rGO/NMC811 cathode. It is well known that graphene sheets aggregate because of the van der Waals forces between them. When preparing the rGO/NMC811 mixture, the aggregation of rGO increases the amount of the few-layers-thick graphene material. Additionally, Li-ions experience higher tortuosity in their movement through material with two-dimensional graphene sheets because the ions cannot travel through the planar graphene structure but instead must travel to the outer edges of the sheets to be able to move past the surface. This increase in tortuosity establishes a diffusion-rate limit in the rGO/NMC811 material that manifests itself as a limitation in the battery’s performance at high C-rates.

**Figure 3:**
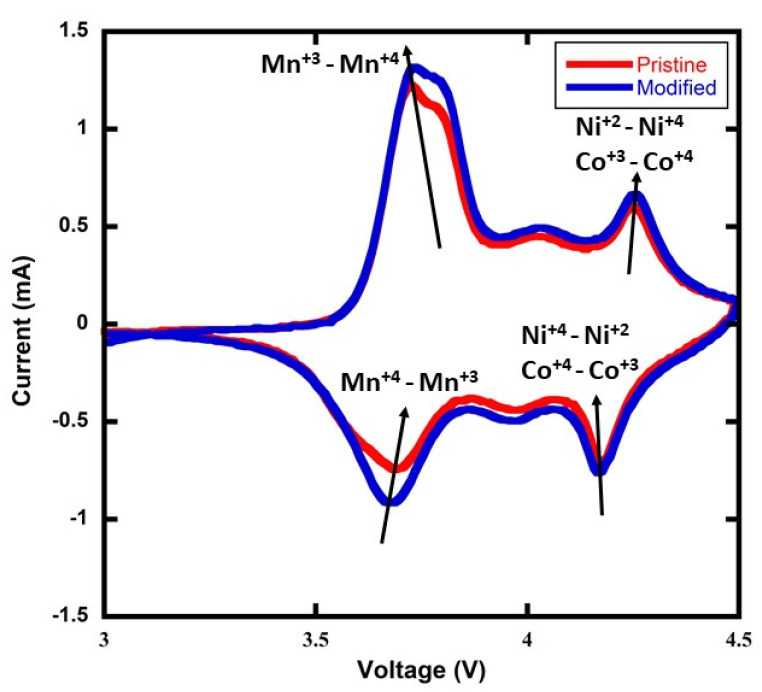
Cyclic voltammetry (CV) measurement for rGO/NMC811 (denoted in the plot legend as modified) with 5wt% rGO additive. The control data are denoted as pristine in the plot legend and show the battery performance with no rGO added. The scan rate is 0.1 mV s${}^{-1}$.

A cyclic voltammetry (CV) measurement was performed to determine whether there was a change in the NMC811 battery’s kinetics after the addition of 5wt% rGO—see Figure 3. The CV plot indicates that there is no change in the Li-ion diffusion or electronic reaction kinetics with the addition of the rGO. However, a small voltage shift ∼20 mV is observed in the plot where the Mn${}^{+4}$− Mn${}^{+3}$ redox reaction is represented. As a proxy for an electrochemical impedance spectroscopy (EIS) measurement that was not available to our group, the CV data are interpreted as suggesting the possibility of a slight increase in the battery cell impedance with the addition of 5wt% rGO. In the future, an EIS measurement will be conducted to further evaluate this observation and to quantify any change in impedance.

**Figure 4:**
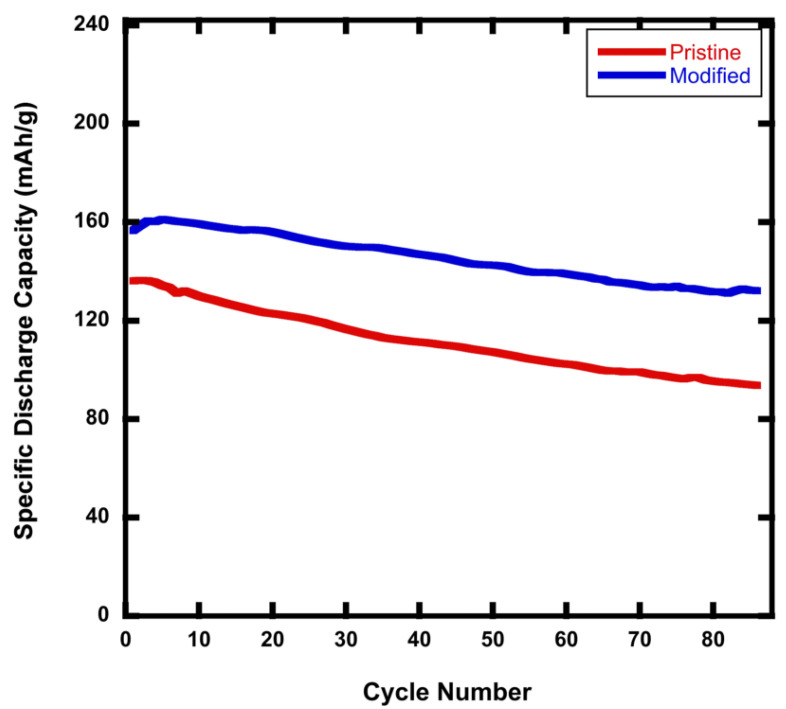
The pristine NMC811 battery has ∼69% capacity retention, whereas the rGO modified battery has ∼85% capacity retention after 88 cycles over the voltage window of 2.5 V–4.5 V at 2C. An additive amount of 5wt% rGO was used in the rGO/NMC811 battery.

On one hand, it is known that NMC811 experiences aggressive capacity fading when operating at high voltage because of side reactions and structural instability [12,26]. On the other hand, cathode structural instability and impedance growth are also accelerated by high C-rates due to an elevated amount of surface delithiation at particle surfaces at the end of charge, as well as the augmented level of mechanical stresses caused by expansions and contractions being repeated at high rates. The modified rGO/NMC811 cathode significantly improves the cyclability of the battery for tests performed over the voltage window of 2.5–4.5 V vs. Li${}^{+}$/Li. Figure 4 shows that, whereas the NMC811 pristine battery had a 69% capacity retention, the modified rGO/NMC811 battery retained about 85% of its capacity after 88 cycles at a C-rate of 2C.

**Figure 5:**
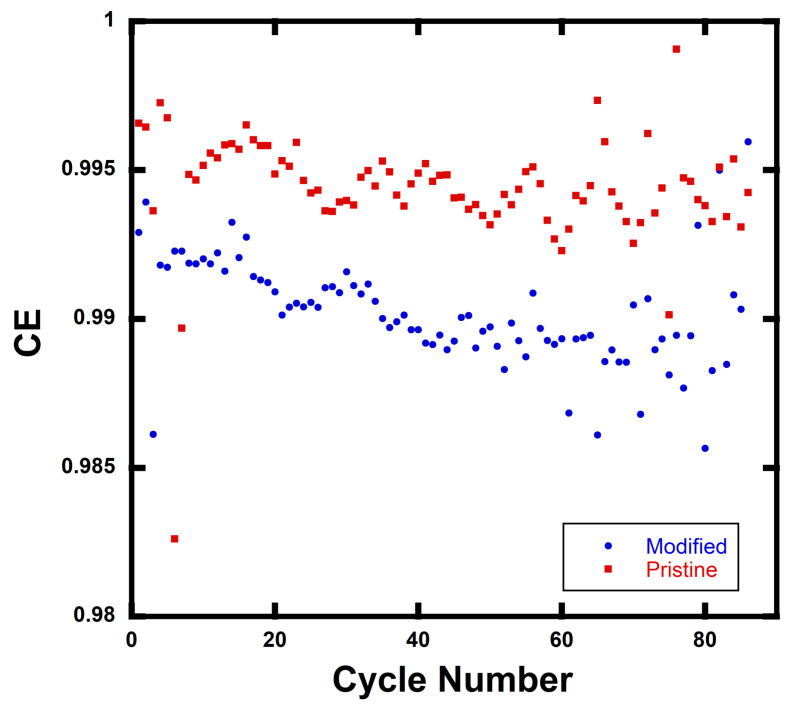
Coulombic efficiency (CE) vs. cycle number for 88 cycles. Pristine represents the control NMC811 battery where no rGO was added. Modified represents the rGO/NMC811 battery with 5wt% rGO. Testing was performed over a voltage window of 2.5–4.5 V at 2C.

Coulombic efficiency (CE) data are shown in Figure 5 for rGO/NMC811 batteries. Each data point is the average of three data points taken for the control batteries (i.e., pristine NMC811 cathode with no added rGO) and rGO/NMC811 batteries with 5wt% rGO. Tests were performed at C-rate = 2C. The CE data indicate the magnitude of side reactions in a battery and are defined as $\eta =C_{d}/C_{c}$, where $C_{d}$ is the discharge capacity of a cell at a single cycle and $C_{c}$ is the charge capacity of the cell in the same cycle. The value η=1 indicates that a cell is totally free of undesired side reactions, and a high CE value typically indicates a long battery lifetime. The average CE, <CE>, for the rGO/NMC811 batteries differs by ∼0.5% from the control NMC811 batteries. $<\mathrm{CE}{>}_{\mathrm{NMC}811}\approx$ 0.995 and $<\mathrm{CE}{>}_{\mathrm{rGO}/\mathrm{NMC}811}\approx$ 0.990 with N = 3 for the calculated <CE> value taken at each cycle. The <CE> remains close to 1 after 88 cycles over the 2.5–4.5 V operating voltage window. Thus, it is concluded that side reactions are either non-existent or minimal.

NMC811 particles are microsized and sphere-like in structure, with nano-sized granules having crystal facets being randomly oriented and densely aggregated. This disordered orientation promotes intergrain stresses at the grain boundaries and invokes deterioration with volume expansions/contractions that are experienced during the lithiation/delithiation processes. This facilitates the development of NMC811 particle cracking, which ultimately leads to inferior electrochemical performance [27,28]. Thus, from the cycle stability test results, we deduce that the addition of rGO retards the degradation (i.e., cracking and deformation), which occurs in NMC811 under high-voltage operation. We hypothesize that the large surface area of rGO flakes enhance the interfacial contact because of their excellent conductivity and the establishment of plane-to-point connections with large numbers of NMC811 particles, which leads to better Li${}^{+}$ and electronic transport through the composite rGO/NMC811 cathode material at low C-rates. To confirm our hypothesis, SEM micrographs are used to make visual observations of the physical condition of the NMC811 particles after battery cycling at high voltage.

**Figure 6:**
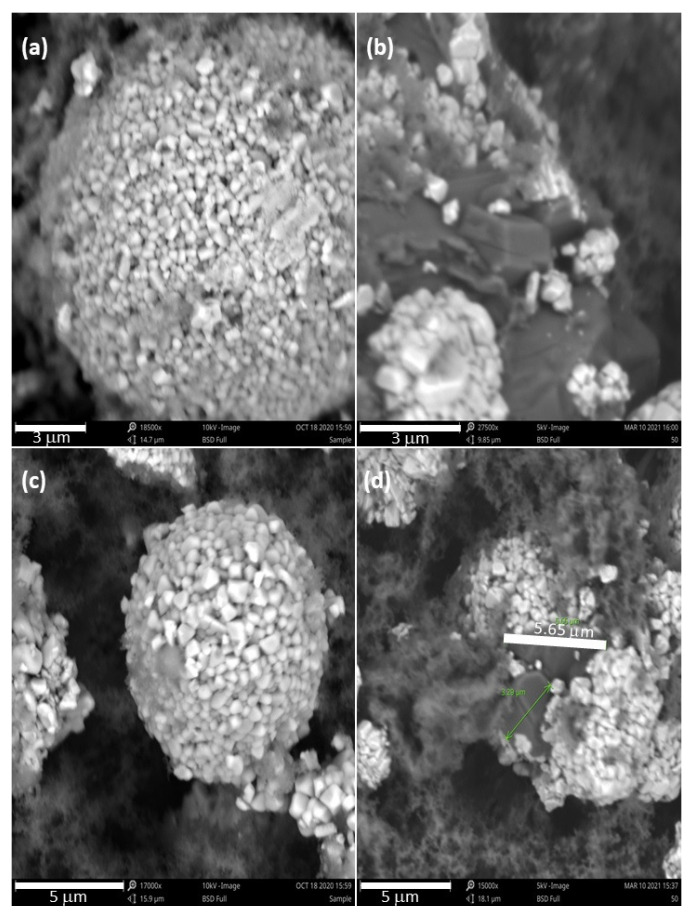
SEM images of pristine NMC811 and rGO/NMC811 cathode material before cycling the batteries. (**a**) A close-up view of pristine NMC811 before battery cycling. Image shows the neatly arranged granules that form a tightly packed NMC811 particle. (**b**) A close-up view of rGO/NMC811 mixture before battery cycling. (**c**) An expanded view of pristine NMC811 before battery cycling. (**d**) An expanded view of rGO/NMC811 before cycling the battery. The amount of rGO additive used was 5wt% of the composite rGO/NMC811 cathode material.

**Figure 7:**
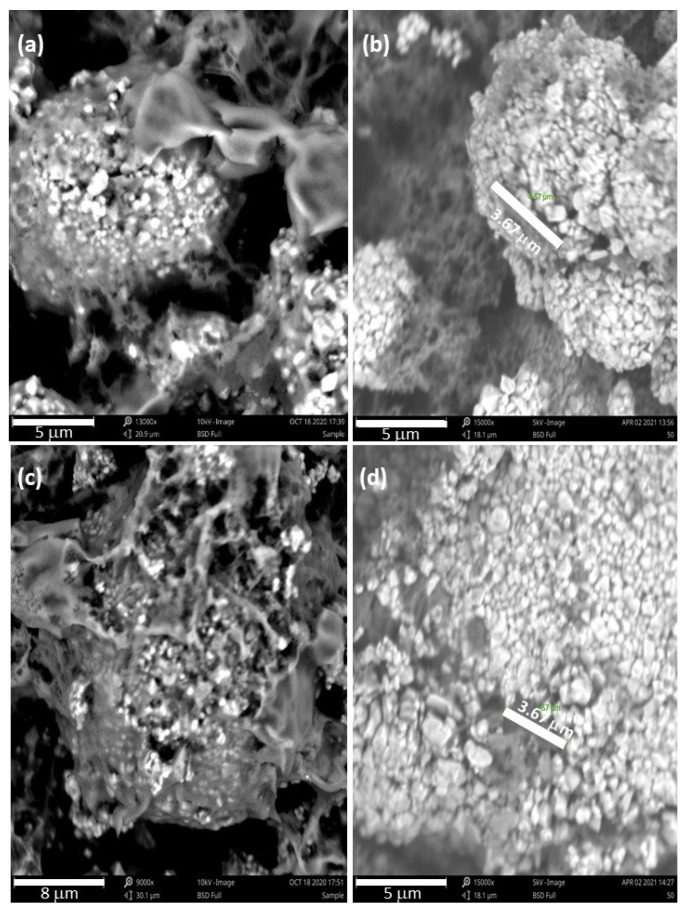
SEM images of pristine NMC811 and rGO/NMC811 cathode material after cycling the batteries for 100 cycles over the high-voltage operating window of 2.5–4.5 V. (**a**) Image showing pristine NMC811 after 100 cycles. Microcracks and missing NMC811 granules can be observed. (**b**) An expanded view of rGO/NMC811 material after 100 cycles. (**c**) A close-up view of the NMC811 material after 100 cycles. (**d**) A close-up view of rGO/NMC811 after 100 cycles. The amount of rGO additive used was 5wt% of the composite rGO/NMC811 cathode material.

Figure 6 and Figure 7 are part of an SEM study that was conducted to see what physical changes the rGO/NMC811 cathode would undergo after cycling at high voltage. The images in Figure 6 were taken before battery operation. Figure 6a,c shows the neatly arranged granules that form tightly packed pristine NMC811 particles. Figure 6b,d shows the rGO/NMC811 cathode material with dark rGO platelets in the space between adjacent bright NMC811 particles. After testing for 88 cycles at 2C over the voltage window of 2.5–4.5 V, batteries were deconstructed for the SEM study. The images in Figure 7 show the cathode material at the conclusion of cycling. Figure 7a,c shows that microcracks formed in the pristine NMC811 particles. After repeated use, battery material loss is observed as missing granules, which manifests itself as capacity fading. However, Figure 7b,d shows that the presence of rGO mitigates the severe degradation of NMC811. The NMC811 particles are in much better condition after 100 cycles under high-voltage operation, due to the rGO additive.

From our observations, rGO material appears to have undergone melting and re-solidification. As the battery operates, local internal temperature hotspots can form within the material at temperatures that may exceed the melting point of graphene oxide. The melting of graphene oxide is consistent with differential scanning calorimetry (DSC) and thermogravimetric analysis (TGA) studies in the literature, which report that flakes of graphene oxide (GO) and reduced graphene oxide (rGO) begin to melt around 50 ${}^{\circ }$C and 110 ${}^{\circ }$C, respectively [29]. Local internal temperature hotspots within Li-ion batteries can exceed these temperatures during operation as electrons move through the cathode material [30]. We conjecture that the re-solidification of melted rGO with NMC811 establishes a type of self-healing mechanism that helps to hold NMC811 particles together after repeated cycling. Maintaining the structural integrity of the NMC811 particles improves their resilience and reduces the growth of charge transfer impedance during electrochemical cycling. Because of the good condition of the NMC811 particles in the rGO/NMC811 after many cycles, capacity fading is prevented from accelerating. Therefore, the rGO/NMC811 battery lifetime is improved.

## 4. Conclusions

In this work, we investigated the performance of NMC811 with rGO additive as a cathode material for high-voltage operation (4.5 V). The rGO/NMC811 cathode composition was studied employing electrochemical testing for discharge capacity performance, C-rate behavior and cyclic voltammetry characteristics; scanning electron microscopy to observe physical changes after prolonged cycling; and cycle testing for battery lifetime performance. C-rate testing included high C-rates up to 5C. A coulombic efficiency study was done to monitor the presence of side reactions that may have occurred. C-rate testing revealed a diffusion-limited C-rate at 3C for the rGO/NMC811 battery. We attribute this to an increase in tortuosity, caused by the rGO additive. Cyclic voltammetry testing showed that the inclusion of 5wt% rGO with NMC811 did not change the NMC811’s charge transfer kinetics. A high coulombic efficiency value after 88 cycles revealed that no significant side reactions occurred in the rGO/NMC811 material. An SEM study of the cathodes taken before and after 100 cycles from 2.5–4.5 V at 2C showed that the rGO/NMC811 material had significantly less degradation than the pristine NMC811. A self-healing mechanism helps to hold NMC811 particles together after repeated cycling. From the SEM study, it appears that aggregates of rGO melt and re-solidify in cracks, thereby helping to reduce the loss of NMC811 granules. We conclude that this mechanism mitigates the spread of microcracks and NMC811 degradation, which reduces the growth of charge transfer impedance during electrochemical cycling. Because of the relatively good condition of the NMC811 particles that can be seen after many cycles under high voltage, capacity fading is prevented from accelerating. Thus, in this study, the rGO/NMC811 cathode with 5wt% rGO experienced ∼17% improvement in capacity retention under high-voltage operation.

## Figures and Tables

**Table 1:** Optimization study for selecting the percentage of reduced graphene oxide (rGO) additive for rGO/NMC811 batteries to operate in a high-voltage window of 2.5–4.5 V. The capacity fade was calculated after 100 cycles.

	Discharge Capacity (mAh g${}^{-1}$)		
rGO (wt%)	@0.1C	@3C	Capacity Fade (%)
0 (control)	202.61	104.81	31
2	193.70	98.00	26
5	216.48	93.20	15
10	153.10	66.55	37

## Data Availability

The data presented in this study are available on request from the corresponding author.
